# Magnesium Inhibits Wnt/β-Catenin Activity and Reverses the Osteogenic Transformation of Vascular Smooth Muscle Cells

**DOI:** 10.1371/journal.pone.0089525

**Published:** 2014-02-25

**Authors:** Addy Montes de Oca, Fatima Guerrero, Julio M. Martinez-Moreno, Juan A. Madueño, Carmen Herencia, Alan Peralta, Yolanda Almaden, Ignacio Lopez, Escolastico Aguilera-Tejero, Kristina Gundlach, Janine Büchel, Mirjam E. Peter, Jutta Passlick-Deetjen, Mariano Rodriguez, Juan R. Muñoz-Castañeda

**Affiliations:** 1 IMIBIC, Hospital Univ Reina Sofia, REDInRen, Cordoba, Spain; 2 Depto. Medicina y Cirugía Animal, University of Cordoba, Cordoba, Spain; 3 Fresenius Medical Care Deutschland GmbH, Bad Homburg, Germany; 4 Department of Nephrology, University of Duesseldorf, Dusseldorf, Germany; 5 Servicio de Nefrologia, Hospital Univ Reina Sofia, REDInRen, Cordoba, Spain; 6 Lipid and Atherosclerosis Unit, IMIBIC/Reina Sofia University Hospital/University of Cordoba, and CIBER Fisiopatologia Obesidad y Nutricion (CIBEROBN), Instituto de Salud Carlos III, Cordoba, Spain; University of California Davis, United States of America

## Abstract

Magnesium reduces vascular smooth muscle cell (VSMC) calcification *in vitro* but the mechanism has not been revealed so far. This work used only slightly increased magnesium levels and aimed at determining: a) whether inhibition of magnesium transport into the cell influences VSMC calcification, b) whether Wnt/β-catenin signaling, a key mediator of osteogenic differentiation, is modified by magnesium and c) whether magnesium can influence already established vascular calcification. Human VSMC incubated with high phosphate (3.3 mM) and moderately elevated magnesium (1.4 mM) significantly reduced VSMC calcification and expression of the osteogenic transcription factors Cbfa-1 and osterix, and up-regulated expression of the natural calcification inhibitors matrix Gla protein (MGP) and osteoprotegerin (OPG). The protective effects of magnesium on calcification and expression of osteogenic markers were no longer observed in VSMC cultured with an inhibitor of cellular magnesium transport (2-aminoethoxy-diphenylborate [2-APB]). High phosphate induced activation of Wnt/β-catenin pathway as demonstrated by the translocation of β-catenin into the nucleus, increased expression of the frizzled-3 gene, and downregulation of Dkk-1 gene, a specific antagonist of the Wnt/β-catenin signaling pathway. The addition of magnesium however inhibited phosphate-induced activation of Wnt/β-catenin signaling pathway. Furthermore, TRPM7 silencing using siRNA resulted in activation of Wnt/β-catenin signaling pathway. Additional experiments were performed to test the ability of magnesium to halt the progression of already established VSMC calcification *in vitro*. The delayed addition of magnesium decreased calcium content, down-regulated Cbfa-1 and osterix and up-regulated MGP and OPG, when compared with a control group. This effect was not observed when 2-APB was added. In conclusion, magnesium transport through the cell membrane is important to inhibit VSMC calcification *in vitro*. Inhibition of Wnt/β-catenin by magnesium is one potential intracellular mechanism by which this anti-calcifying effect is achieved.

## Introduction

Clinical observations in uremic patients show that high phosphate is associated with an increase in mortality [1]–[4]. Results obtained from experimental work demonstrate that high serum phosphate is a critical factor in the development of vascular calcification [3], [5]. Thus phosphate binders that help to control serum phosphate are commonly used in uremic patients [6]–[8].

Clinical studies indicate that magnesium-containing phosphate binders are effective in controlling serum phosphate [9]–[12], and their phosphate lowering properties have been described as either resulting in or being associated with a decrease in vascular calcification [13], [14].

Dialysis patients tend to have increased concentrations of serum magnesium and those patients receiving magnesium-containing phosphate binders show a further elevation that is moderate but significant, albeit not associated with any clinical symptoms [9]. The control of serum phosphate achieved with the use of magnesium-based phosphate binders should benefit vascular calcification; however, the impact of a moderate increase in serum magnesium on vascular calcification is not clear and therefore deserves attention. Experimental studies suggest that a high concentration of magnesium (2 to 3 mM) may prevent calcification *in vitro* [15]–[18]; In addition, an interventional animal study revealed reduced vascular calcification upon magnesium-containing phosphate binder treatment [19]. However, the magnesium concentrations used in these studies were much higher than the serum magnesium levels found in patients treated with magnesium-containing phosphate binders (1.3±0.3 mM or 1.0±0.1 mM) [9], [20].

The mechanisms by which magnesium may influence vascular calcification are unclear. It is known that *in vitro*, magnesium impairs hydroxyapatite crystal growth [21]. A recent work [17] demonstrated that inhibition of magnesium channels in VSMC cultured with high phosphate and magnesium results in changed expression of osteogenic genes. However, the influence of cellular magnesium transport on vascular calcification at slightly elevated concentrations and the mechanisms involved remain to be elucidated.

Wnt signaling, which is essential for the osteogenic commitment of pluripotent mesenchymal cells, has also been shown to be activated during the development of vascular calcification *in vivo* and *in vitro* [22]–[27]. Wnt proteins are a large family of secreted molecules that signal through binding to a co-receptor complex formed by proteins of the frizzled family and the lipoprotein receptor-related 5/6 proteins. The activation of the canonical Wnt pathway results in the nuclear translocation of β-catenin and subsequent regulation of target gene expression [27].

The present study using only slightly elevated magnesium concentrations was performed to determine, a) whether magnesium transport into the cell has any influence on VSMC calcification b) whether magnesium modifies Wnt/β-catenin signaling and c) whether magnesium has an influence on already established VSMC calcification.

## Methods

### VSMC culture

Human aortic smooth muscle cells (VSMC) were obtained from Clonetics (Lonza Walkersville, Inc., USA). Cells were cultured in DMEM supplemented with FBS (20%) (Bio Whittaker; Verviers, Belgium), Na pyruvate (1 mM), glutamine (4.5 g/L), penicillin (100 U/mL), streptomycin (100 mg/mL), and HEPES (20 mM) (all reagents from Sigma Aldrich Inc; MO, USA) at 37°C in a humidified atmosphere with 5% CO_2_. Cells were used after the 5th passage.

### Experimental design

In the control groups VSMC were cultured with standard magnesium concentration (0.8 mM) and phosphate concentration (0.9 mM). In the high phosphate groups, cells were incubated with medium described above supplemented with phosphate salts: Na_2_HPO_4_ and NaH_2_PO_4_ in 1∶2 proportion (Sigma Aldrich Inc; MO, USA), to reach a final concentration of 3.3 mM phosphate. In the high magnesium groups MgCl_2_ was added to the high phosphate medium to reach a final concentration of 1.4 or 2.6 mM. Three assays were performed with VSMC: a) to confirm the effect of magnesium on VSMC calcification (9 days culture with the same magnesium level), b) to study early expression of genes implicated in Wnt/β-catenin signaling pathway (24 hours in culture) and c) to reverse VSMC calcification (adding magnesium at the 5^th^ day of culture in calcifying conditions).

### TRPM7 inhibition by 2-APB

Transient Receptor Potential Melastin 7 (TRPM7) was inhibited in VSMC by incubation with 10 µM of 2-aminoethoxy-diphenylborate (2-APB, Sigma-Aldrich Inc; MO, USA).

### Quantification of calcification

After the incubation period, cells were decalcified by 24 h incubation in HCl (0.6 M). The calcium content in the supernatant was determined by the phenolsulphonephthalein method (QuantiChrom™ Calcium Assay Kit, BioAssay Systems, CA, USA). Cells were washed 3 times with PBS (Sigma Aldrich Inc; MO, USA) and solubilized in 0.1 M NaOH/0.1% SDS. Cell protein content was measured by Bio-Rad protein assay (Bio-Rad Laboratories GmbH, Munich, Germany). The calcium content was normalized for total protein.

### Real-Time polymerase chain reaction (RT-PCR)

Total RNA was isolated from VSMC using a RNA extraction kit (Rneasy™, Qiagen, USA). cDNA was synthesized with a first strand cDNA synthesis kit (Roche; IN, USA) from 0.5 µg of total RNA. The primers used for PCR amplification are shown in Table 1. Real-time PCR was performed in duplicate with QuantiTect SYBR Green PCR (Qiagen; USA) according to the manufacturer's protocol. All PCR amplifications were carried out using Lightcycler (Roche Molecular Biochemicals; IN, USA.). The expression of target genes was normalized to the expression of β-actin. All primers used were equally efficient.

**Table 1:** Primers used for quantitative RT-PCR analyses.

Cbfa-1	Fw	5′-GCA GTT CCC AAG CAT TTC AT-3′
	Rv	5′-CGG ACA TAC CGA GGG ACA T-3′
Osterix	Fw	5′-ATC TGC CTG CGT CCT TGG GAC CCG-3′
	Rv	5′-TGC TTT GCC CAG AGT TGT TGA GTC-3′
MGP	Fw	5′-CCT GAA GTA GCG ATT ATA GGC-3′
	Rv	5′-CCT GAA GTA GCG ATT ATA GGC-3′
OPG	Fw	5′-CTT CAG GTT TGC TGT TCC TAC-3′
	Rv	5′-CAG AGG TCA ATA TCT TGG ATG-3′
Frizzled 3	Fw	5′-TGG AGC CAT TCC ACC CTA TG-3′
	Rv	5′-GAA CCT ACT GCA TTC CAT ATC-3′
VCAN/versican	Fw	5′-ATA CGT GCA AGA AAG GAA CAG TCG-3′
	Rv	5′-GTC CTT TGG TAT GCA GAT GGG TTC-3′
Cyclin D1	Fw	5′-CCG AGG AGC TGC TGC AAA TGG A-3′
	Rv	5′-ATG GAG GGC GGA TTG GAA ATG AAC-3′
c-Myc	Fw	5′-ACC ACC AGC AGC GAC TCT GAG GA-3′
	Rv	5′-CGT AGT TGT GCT GAT GTG TGG AGA-3′
DKK-1	Fw	5′-ATA TTC CAG CGT TGT TAC TGT-3′
	Rv	5′-CCA CAC TGA GAA TTT ACA ATA C-3′
β-actin	Fw	5′-GCA CTC TTC CAG CCT TCC TT-3′
	Rv	5′-ATC CAC ATC TGC TGG AAG GT-3′

### Confocal microscopy

VSMC were seeded on coverslips, and after reaching 90% confluence, they received the different treatments for 24 h. Then, they were rinsed in PBS, fixed and permeated in cold 50% methanol for 2 min, cold 100% methanol for 20 min, and cold 50% methanol for 2 min. The specimens were subsequently washed in PBS (3×5 min) and incubated for 1 h with anti-β-catenin antibody (1∶50; BD Pharmigen, NJ, USA) in blocking solution (1% BSA) at room temperature. After being washed with PBS (3×5 min), specimens were incubated for 1 h with Alexa Fluor 488 F(ab')_2_ fragment of rabbit anti-mouse IgG (1∶5000; Invitrogen, Paisley, UK) in PBS containing 1% BSA. After a final wash with PBS (3×5 min), the specimens were counterstained with DAPI for nuclear stain. Cells were mounted on slides to examine fluorescence using a LSM 5 Exciter Carl Zeiss confocal microscope. ImageJ software (National Institute of Health, USA) was used to analyze confocal immunofluorescence staining. Mander's coefficient M2 plugin (DAPI vs. green) was used to analyze nuclear translocation of β-catenin. Mander's coefficient M2 is the percentage of above-background pixels in blue color (DAPI) that overlap above-background pixels in green color (β-catenin).

### siRNA knockdown of TRPM7 expression

A small interfering RNA (siRNA) was generated against TRPM7. siRNA for knocking down TRPM7 was synthesized by Qiagen (USA). The DNA target sequence of the annealed double strand siRNA that we used was: 5′-CCT GTA AGA TCT ATC GTT CAA-3′. siRNA, with a nonsilencing oligonucleotide sequence (nonsilencing siRNA) that does not recognize any known homology to mammalian genes, was also generated as a negative control.

Cells (VSMCs) were seeded at a density of 2×10^5^ cells/well in 6-well plates and grown in DMEM containing 15% FBS and antibiotics. One day after seeding, cells were transfected with siRNA using siRNA Reagent System (Santa Cruz Biotechnology Inc, USA) according to the manufacturer's instructions. Briefly, siRNA (40 pmol) was mixed with transfection medium (100 µL) to which transfection reagent (6 µL) plus transfection medium (100 µL) was added. After mixing (45 minutes), the formulation was added dropwise onto the cells. Control cells were exposed to transfectant in the absence of siRNA. 18 hours after transfection, media was changed to DMEM containing 15% FBS and antibiotics. Cells were lysed after 24 hours, and gene silencing was monitored at the mRNA level by RT-PCR.

### Statistics

Data are expressed as mean±SE. The difference between means from two different groups was evaluated by t-test; the difference between means for three or more groups was assessed by ANOVA followed by Bonferroni post hoc analysis. P values less than 0.05 were considered significant. The analyses were performed using SPSS 15.0.

## Results

### The effect of magnesium on vascular smooth muscle cell calcification

**Figure 1:**
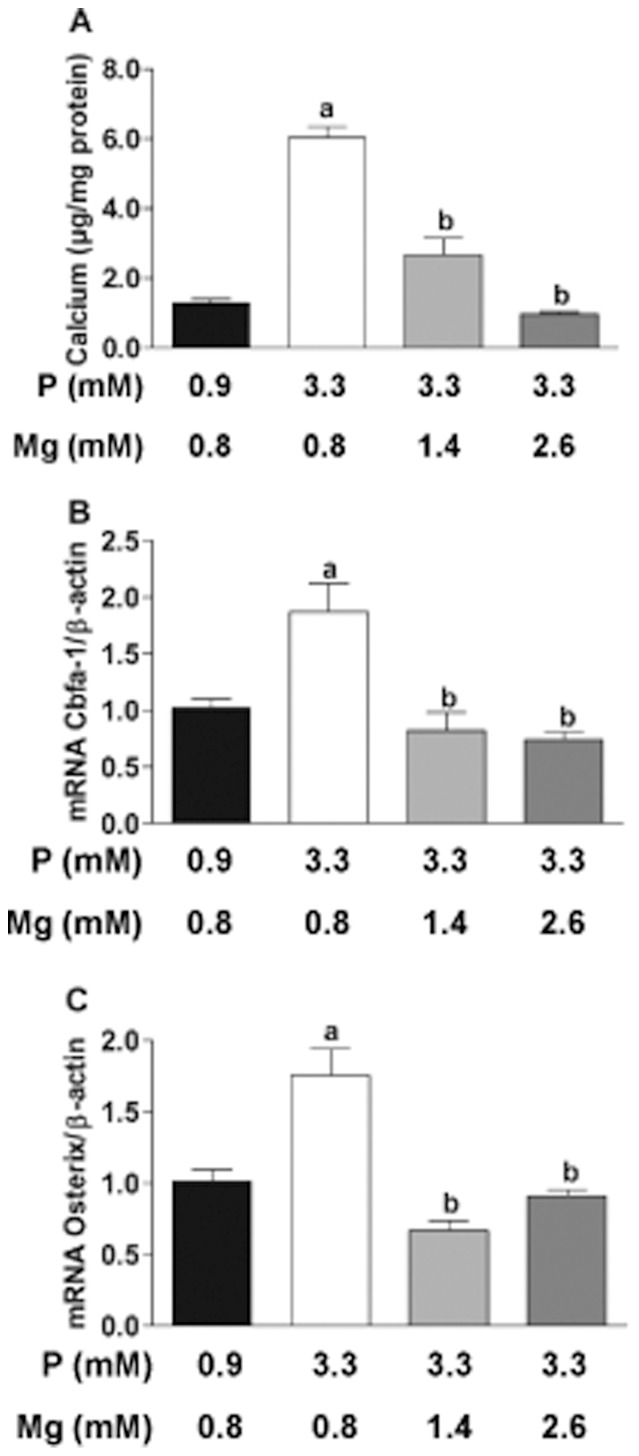
The effect of magnesium on calcification and osteoblast-like transformation in VSMC *in vitro*. VSMC were cultured with 3.3 mM P, and increasing concentrations of magnesium (0.8 mM/1.4 mM/2.6 mM); control group with 0.9 mM P+0.8 mM Mg was also included. (**A)**, calcium content of VSMC cultured for 9 days. Relative mRNA expression of Cbfa-1 **(B)** and osterix **(C)**; **a**, P<0.001 vs Control; **b**, P<0.001 vs VSMC with 3.3 mM P+0.8 mM Mg. Bars are the mean±SE of three experiments in quadruplicate.

Incubation of VSMC in a medium with high phosphate (3.3 mM) and physiological magnesium (0.8 mM) concentrations produced calcification when compared to controls (p<0.001) (Figure 1A). The addition of magnesium to the medium up to 1.4 mM caused a significant (p<0.001) reduction in calcification. With a magnesium concentration of 2.6 mM, calcium content was reduced further, which was similar to the amount of calcium found in the control group.

Gene expression of the central osteogenic transcription factor Cbfa-1 as well as its downstream effector osterix was significantly increased in VSMC incubated with high phosphate medium vs. controls (Figures 1B and 1C). The expression of Cbfa-1 and osterix was reduced to control levels by the addition of magnesium to 1.4 mM. No further reduction in gene expression of osteogenic transcription factors was detected when magnesium concentration was increased to 2.6 mM.

### Effect of inhibition of cellular magnesium receptor (TRPM7) on VSMC calcification and osteogenic gene expression

**Figure 2:**
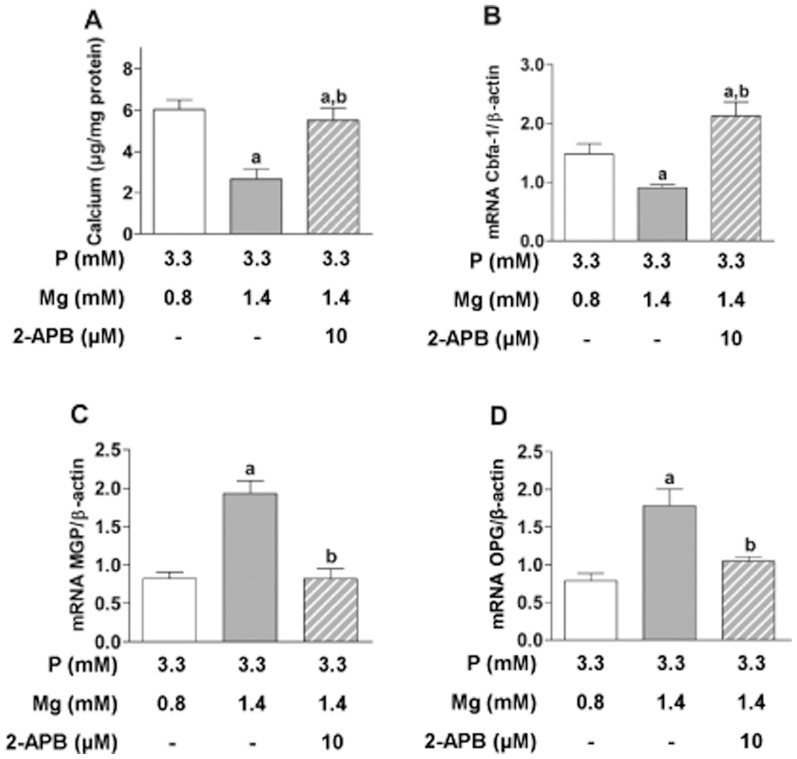
Inhibition of TRPM7 abrogates protective effect of magnesium on calcification and gene expression on VSMC. VSMC were cultured with 3.3+1.4 mM Mg with the TRPM7 inhibitor 2-APB (10 µM). VSMC with 3.3 mM P+0.8 mM Mg were also included. The graphics are **(A)** calcium content, and the relative mRNA expression of **(B)** Cbfa-1, **(C)** MGP, and **(D)** OPG. **a**, P<0.05 vs VSMC with 3.3 mM P+0.8 mM Mg; **b**, P<0.05 vs VSMC with 3.3 mM P+1.4 mM Mg. Bars are the mean±SE of three experiments in quadruplicate.

After having demonstrated the protective effect of magnesium on VSMC calcification, further experiments were performed, and the effect of 2-APB addition, an inhibitor of the magnesium receptor TRPM7, was investigated. As shown in Figure 2A, the protective effect of magnesium was abolished: Despite a magnesium concentration of 1.4 mM, the addition of 10 µM 2-APB increased calcification to the same level as observed with a magnesium concentration of 0.8 mM (p<0.001) (Figure 2A).

The increase in calcification observed with 10 µM 2-APB was accompanied by an intensification of Cbfa-1 gene expression (Figure 2B) as compared to VSMC incubated with the same medium (3.3 mM phosphate/1.4 mM magnesium) but without 2-APB (p<0.001).

To confirm the downstream effect the expression of matrix Gla protein (MGP) (Figure 2C), a naturally occurring calcification inhibitor [28], was investigated. It was increased (p<0.001) by the high magnesium concentration (1.4 mM) despite the presence of high phosphate, as compared to VSMC exposed to 3.3 mM phosphate/0.8 mM magnesium. However, the addition of 10 µM 2-APB reduced MGP expression to the level obtained with a magnesium concentration of 0.8 mM.

Low osteoprotegerin (OPG) has been associated with vascular calcification [29]. A magnesium concentration of 1.4 mM doubled OPG expression (Figure 2D) as compared with the 0.8 mM magnesium condition. However, the addition of 10 µM 2-APB prevented the increase in OPG level induced by magnesium (p<0.005).

### Magnesium inhibits phosphate-induced Wnt/β-catenin signaling

**Figure 3:**
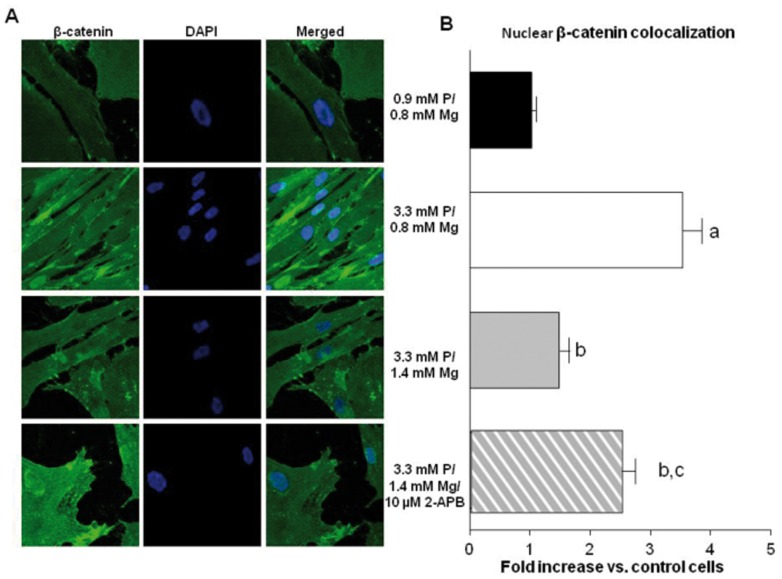
Magnesium inhibits β-catenin translocation into the nucleus in VSMC. VSMC cultured with 3.3+1.4 mM Mg, and 3.3 mM P+1.4 mM Mg+10 µM 2-APB. Control group with 0.9 mM P+0.8 mM Mg was also included. Intracellular localization of β-catenin was visualized by immunofluorescence using confocal microscopy. **(A)** For each group, β-catenin (green immunofluorescence) is shown left; in the middle the same sample is counterstained with DAPI (blue) for nuclear stain; the merged image is shown right. Images represent four different experiments. **(B)** Quantification of nuclear β-catenin staining was performed by the Mander's coefficient (M2 plugin: DAPI vs. green). **a**, P<0.005 vs controls; **b**, P<0.005 vs VSMC with 3.3 mM P+0.8 mM Mg; and **c**, P<0.05 vs VSMC with 3.3 mM P+1.4 mM Mg. Bars are the mean±SE of four experiments.

The Wnt/β-catenin signaling pathway is known to be important in phosphate-induced VSMC calcification (26), and its activation results in nuclear translocation of β-catenin. The amount of intracellular β-catenin was visualized by immunofluorescence (Figure 3A). Cells cultured at normal phosphate and magnesium concentrations showed nuclear exclusion of β-catenin whereas cells cultured with high phosphate/normal magnesium concentrations presented a marked nuclear presence of β-catenin. An increase in magnesium to 1.4 mM prevented nuclear translocation of β-catenin. This effect was no longer observed after the addition of 10 µM 2-APB which blocks magnesium's entry via TRPM7. Quantification of nuclear β-catenin staining intensity (see methods) confirmed the differences in the levels of nuclear β-catenin fluorescence between groups (Figure 3B).

**Figure 4:**
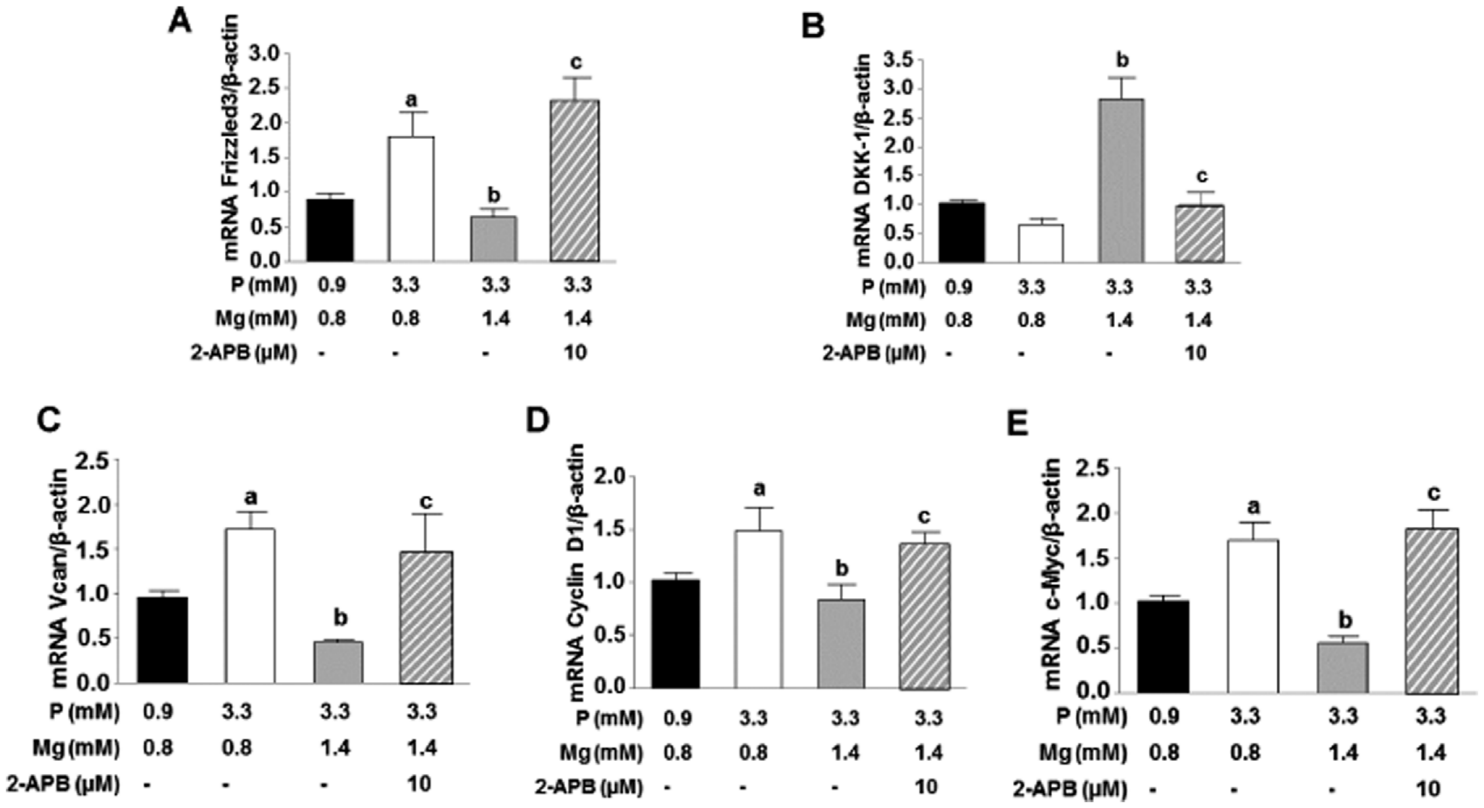
Magnesium inactivates Wnt/β-catenin signaling pathway in VSMC. Relative mRNA expression on VSMC cultured with 3.3+1.4 mM Mg, and 3.3 mM P+1.4 mM Mg+10 µM 2-APB. Control group with 0.9 mM P+0.8 mM Mg was also included. **(A)** Frizzled 3, **(B)** DKK-1, **(C)** VCAN/versican, **(D)** cyclin D1, and **(E)** c-Myc. **a**, P<0.05 vs controls; **b**, P<0.05 vs VSMC with 3.3 mM P+0.8 mM Mg; and **c**, P<0.05 vs VSMC with 3.3 mM P+1.4 mM Mg. Bars are the mean±SE of three experiments in triplicate.

The expression of genes involved in Wnt/β-catenin signaling pathway were also evaluated. Gene expression of frizzled 3 (Figure 4A), a receptor for Wnt ligands, was enhanced in VSMC treated with high phosphate/normal magnesium as compared to cells incubated with normal phosphate/normal magnesium. Increasing the magnesium concentration to 1.4 mM decreased the expression of frizzled 3 to values observed in cells incubated at control conditions. The addition of 10 µM 2-APB reversed this effect and frizzled 3 expression increased to a level also detected under conditions with high phosphate/normal magnesium.

Dkk-1 (Dickkopf-related protein 1) is a specific natural antagonist of the Wnt/β-catenin pathway. It non-significantly decreased in VSMC cultured with high phosphate/normal magnesium (Figure 4B), while VSMC incubated with high phosphate/high magnesium showed a marked increase in Dkk-1 expression. The addition of 10 µM 2-APB reversed the effect of high magnesium on Dkk-1 expression.

As additional direct Wnt/β-catenin transcription targets VCAN/versican (a large chondroitin sulfate proteoglycan; a member of the aggrecan/versican proteoglycan family. Figure 4C), cyclin D1 (an early marker of cells entering the cell cycle. Figure 4D) and c-Myc (a transcription factor which plays a direct role on DNA replication. Figure 4E) were examined. High phosphate/normal magnesium medium enhanced the expression of VCAN/versican, cyclin D1 and c-Myc, whereas 1.4 mM magnesium significantly decreased the expression. This effect was reversed by inhibition of magnesium transport using 2-APB.

### Knock down of TRPM7 activates Wnt/β-catenin signaling

Treatment with 2-APB suggested that TRPM7 was involved in the regulation of Wnt pathway by TRPM7 channels, however the actions of 2-APB are not restricted to inhibition of TRPM7. To confirm the specific role of TRPM7 in the regulation of canonical Wnt signaling, human VSMC were transfected to knock down TRPM7 expression.

**Figure 5:**
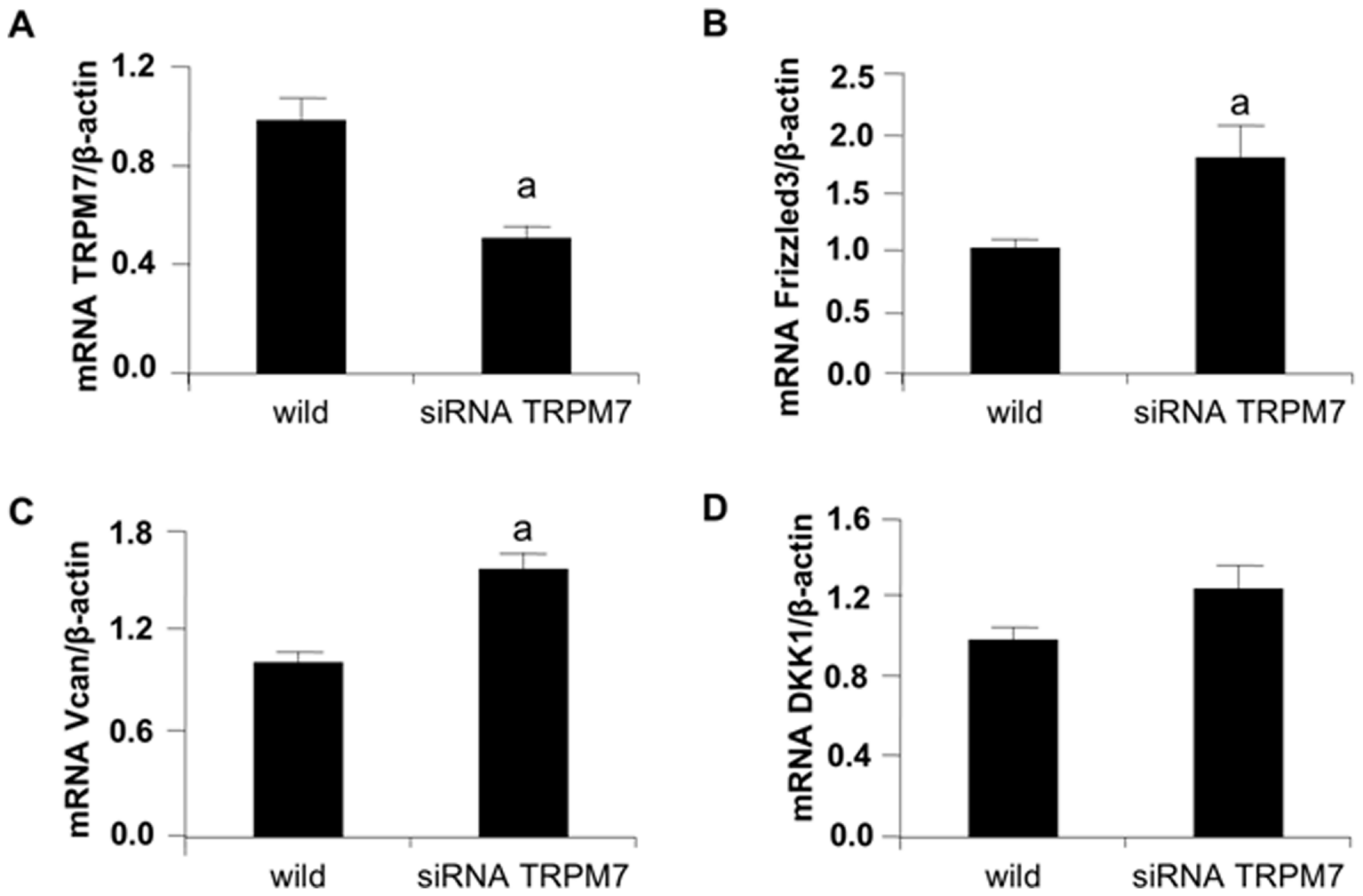
siRNA inhibition of TRPM7 activates Wnt/β-catenin signaling pathway in VSMC. Relative mRNA expression of VSMC transfected with siRNA of TRPM7 24-type VSMC were included as controls. **(A)** TRPM7, **(B)** Frizzled 3, **(C)** VCAN/versican, **(D)** DKK-1. **a**, P<0.05 vs controls. Bars are the mean±SE of two experiments in triplicate.

TRPM7 mRNA expression was 50% reduced (p<0.001) in VSMC transfected with siRNA vs. wild-type cells (Figure 5A). Gene expression of frizzled 3 receptor was significantly (p<0.05) increased on transfected VSMC (Figure 5B). Indeed, transfection with TRPM7 siRNA enhanced expression of the direct Wnt/β-catenin transcription target VCAN/versican (p<0.005; Figure 5C). No differences were observed between transfected and not transfected cells in DKK1 expression (Figure 5D).

### Magnesium halts and decreases calcification in cultured vascular smooth muscle cells

To investigate the effect of magnesium on already existing calcification, human VSMC were incubated with high phosphate and normal magnesium concentrations in the medium during a 5 day period and then switched to one of the following culture media for an additional 4 days: a) medium with high phosphate and high magnesium (1.4 mM), b) medium with high phosphate and high magnesium supplemented with 10 µM 2-APB, and c) medium with normal phosphate and normal magnesium; this last medium served to compare the benefits of lowering phosphate concentration versus increasing magnesium concentration. Some cells were maintained in high phosphate and normal magnesium until day 9.

**Figure 6:**
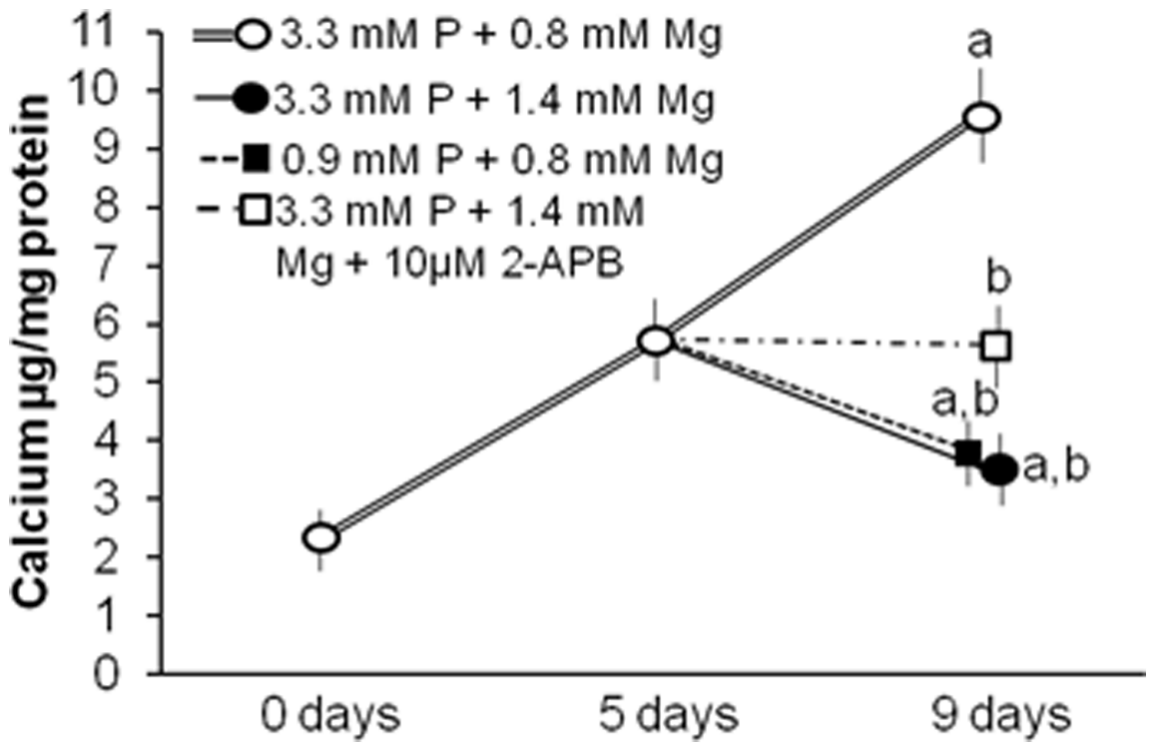
Magnesium halts VSMC calcification induced by high phosphate. VSMC calcification was induced with 3.3+0.8 mM Mg for 5 days. At the 5^th^ day, the medium was switched to 3.3 mM P+1.4 mM Mg, 3.3 mM P+1.4 mM Mg+10 µM 2-APB or 0.9 mM P+0.8 mM Mg and was maintained until day 9; **a**, P<0.01 vs 0 days; **b**, P<0.05 vs VSMC with 3.3 mM P+0.8 mM Mg for 5 days; **c**, P<0.001 vs VSMC with 3.3 mM P+0.8 mM Mg for 9 days. Values are the mean±SE of four experiments in quadruplicate.

In VSMC cultured at normal magnesium and high phosphate, calcification progressively increased until the end of the experiment at day 9 (Figure 6). The increase in magnesium from 0.8 to 1.4 mM at day 5 produced a subsequent decrease in calcification (p<0.01). The addition of 10 µM of 2-APB to the high phosphate/high magnesium media at day 5, produced no significant changes in calcium content from day 5 to day 9.

Lowering phosphate to normal concentrations at day 5 resulted in a significant decrease in calcification at day 9 (p<0.05). This effect was similar to the one observed in the 3.3 mM phosphate/1.4 mM magnesium group.

**Figure 7:**
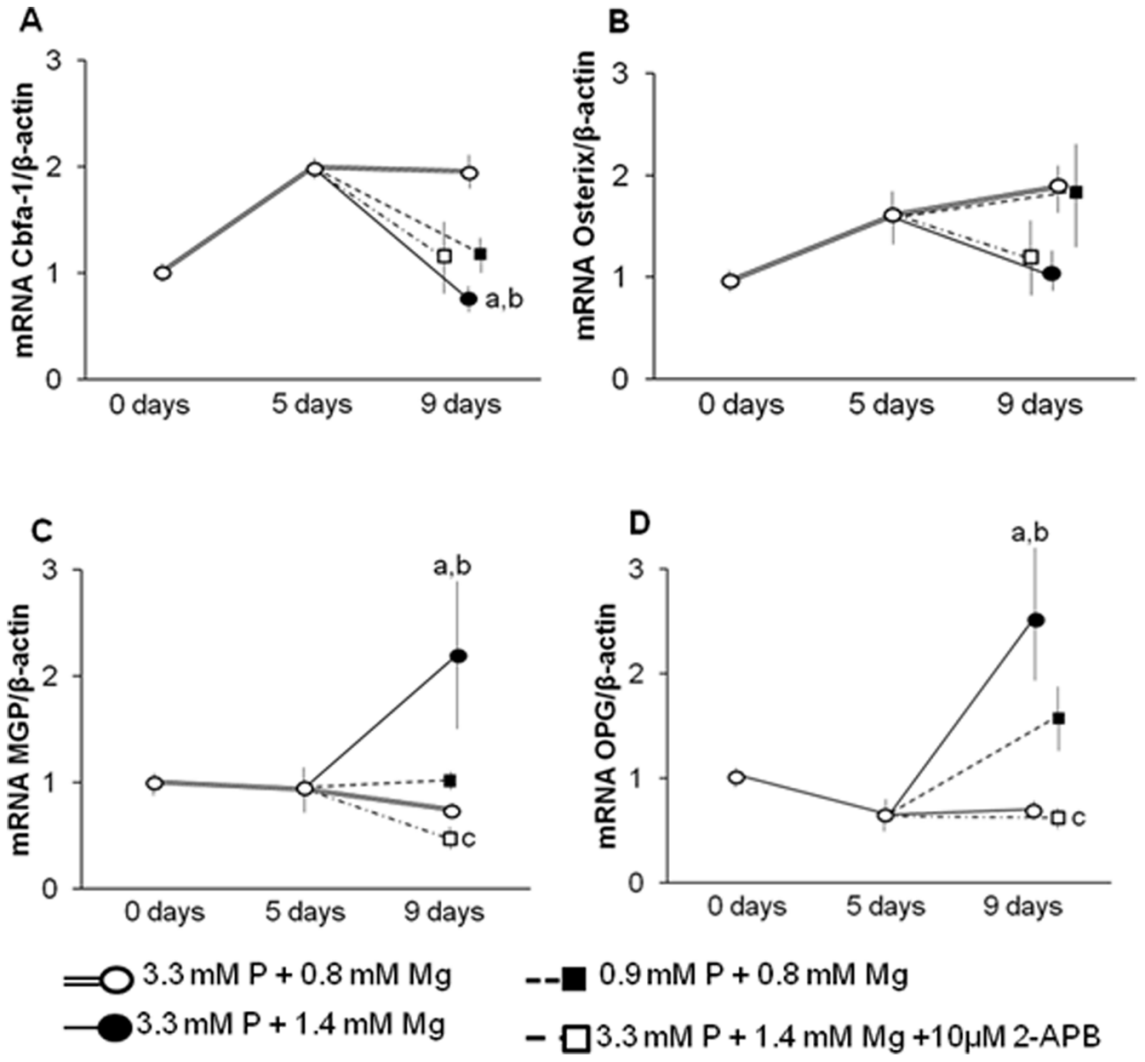
Magnesium halts Cbfa-1 expression and up-regulates MGP and OPG when added to VSMC with already existing calcification. VSMC calcification was induced with 3.3+0.8 mM Mg for 5 days. At the 5^th^ day, the medium was switched to 3.3 mM P+1.4 mM Mg, 3.3 mM P+1.4 mM Mg+10 µM 2-APB or 0.9 mM P+0.8 mM Mg and was maintained until day 9. The graphics are the relative mRNA expression of **(A)** Cbfa-1, **(B)** osterix, **(C)** MGP, and **(D)** OPG. **a**, P<0.05 vs VSMC with 3.3 mM P+0.8 mM Mg for 5 days 0 days; **b**, P<0.05 vs VSMC with 3.3 mM P+0.8 mM Mg for 9 days; and c, P<0.01 vs VSMC with 3.3 mM P+1.4 mM Mg for 5 to 9 days. Values are the mean±SE of three experiments in triplicate.

As shown in Figure 7A, the expression of Cbfa-1 was increased at day 5 (p<0.001) in VSMC treated with high phosphate/normal magnesium and remained elevated until day 9. Adding 1.4 mM magnesium at day 5 significantly (p<0.01) decreased Cbfa-1 expression at day 9. Treatment with 10 µM 2-APB showed a tendency (n.s.) to increase Cbfa-1 expression. Similar results were observed when cells were switched to normal phosphate/normal magnesium medium at day 5: Cbfa-1 expression was slightly enhanced as compared to the 1.4 mM magnesium group (n.s.).

In these experiments the expression of osterix (Figure 7B) showed the same tendency as the Cbfa-1 expression, but intergroup differences were not significant.

MGP expression (Figure 7C) was up-regulated by the addition of magnesium at day 5 and 2-APB caused a down-regulation of MGP. A reduction of phosphate concentration to 0.9 mM did not produce any change in MGP expression.

Similar results were observed in OPG expression (Figure 7D): in cells incubated at high phosphate, the increase in magnesium to 1.4 mM at day 5 produced a significant increase in OPG expression by day 9. However, OPG expression was significantly down-regulated by 2-APB. Lowering phosphate to normal levels slightly increased OPG expression (n.s.).

## Discussion

The present work confirms that magnesium reduces VSMC calcification and osteogenic transdifferentiation even at only slightly elevated, clinically relevant magnesium concentrations. This effect is mediated through cellular magnesium transport as an inhibition of the magnesium transporter TRPM7 results in the abolition of the beneficial effects of magnesium on calcification and prevention of osteogenic phenotype. Furthermore, this study provides insights into intracellular mechanisms for the first time. It shows that magnesium down-regulates the Wnt/β-catenin signaling pathway which may explain its protective effect against VSMC calcification. Interestingly, magnesium not only reduces calcification of VSMC, but is also able to reverse this process after it has been initiated. Taken together our findings demonstrate that magnesium, even at moderately elevated concentrations, has an active role in the prevention of phosphate-induced VSMC calcification.

The present study used an *in vitro* model of VSMC calcification that has been widely applied [30]–[33]. In this model the presence of high phosphate produces osteogenic differentiation and calcification of VSMC.

Recent *in vitro* studies have demonstrated the benefits of magnesium on vascular calcification and provided important insights into magnesium's role in regulating this process. Magnesium concentrations of 2 to 3 mM have been shown to reduce calcification and osteogenic transformation of VSMC [15]–[18]. However, these magnesium concentrations are higher than the values observed in patients taking magnesium-based phosphate binders (1 to 1.4 mM) [9], [11], [20]. Our study used 1.4 mM magnesium and was chosen to mimic a level closer to the one observed in patients.

Our results show that 1.4 mM magnesium substantially decreases calcification and osteogenic transdifferentiation in VSMC incubated with high phosphate.

Furthermore, we found that the osteogenic transcription factors Cbfa-1 and osterix are decreased while the expression of both natural calcification inhibitors MGP and OPG are increased. Down-regulation of Cbfa-1 and up-regulation of MGP by magnesium has been previously described in VSMC [15], [17] but to our knowledge, the association between magnesium and osterix as well as OPG in the context of VSMC calcification has not been reported so far.

Osterix is a transcription factor influencing the maturation of osteoblasts and has shown to be elevated in calcifying VSMC [34]. OPG is a protein which is expressed in normal VSMC and down-regulated in calcified VSMC [29]. This protein protects the cells against calcification by reducing alkaline phosphatase activity [35], as well as by exerting an inhibitory effect on apoptosis [36]. This is important as apoptotic bodies may act as nucleation sites for the crystallization of apatite [37], [38]. Moreover, a recent study showed that magnesium at a concentration of 2–3 mM inhibits high phosphate-induced apoptosis [15].

Despite these different investigations the mechanism(s) by which magnesium reduces vascular calcifications are still not fully elucidated. It has been shown that magnesium influences calcium/phosphate (hydroxyapatite) crystallization [39]. Even at low concentrations, magnesium ions have a marked effect on nucleation and growth of calcium phosphates. These ions delay the conversion of amorphous calcium precipitates to the more stable apatite phase and promote the formation of whitlockite [21], [40]–[42]. Whitlockite is a calcium/magnesium orthophosphate (Ca,Mg)_3_(PO4)_2_ that may produce less stress in VSMC than pure hydroxyapatite crystals. In addition to this passive phenomenon, these and other results also point to an active role of magnesium and a direct effect on gene expression [16].

To test if the observed effect of magnesium in preventing calcification requires active transport of magnesium into the cells, VSMC were exposed to 2-APB, an inhibitor of TRPM7 which regulates magnesium homeostasis in VSMC [17], [43], [44]. The results of our experiments are uniform: an inhibition of magnesium transport completely abolishes the beneficial effects of magnesium on VSMC calcification. The central osteogenic transcription factor Cbfa1 is upregulated in VSMC cultured with high phosphate, magnesium and 2-APB, indicating that the inhibitory effect of magnesium on phosphate-induced overexpression of this gene is no longer present. Furthermore, the preventive effect of magnesium on the reduced gene expression of the effectors MGP and OPG in VSMC under calcifying conditions is abrogated in cultures exposed to the TRPM7 inhibitor. These findings suggest that in addition to the above mentioned effects of magnesium on crystal growth there seems to be an intracellular effect of magnesium on the regulation of calcification and osteoblast-like transformation. This effect depends on an active entry of magnesium via TRPM7. As a unique characteristic the “chanzymes” TRPM7 and its homologue TRPM6 also possess an intracellular alpha-kinase domain [44]. Its influence on the transporter activity has been shown for TRPM6 [45] and therefore might as well affect the processes observed here.

Wnt/β-catenin pathway has been implicated in the regulation of phosphate-induced osteogenic transdifferentiation and calcification in VSMC *in vitro* [25], [27], [46], [47]. We show that in VSMC cultured with high phosphate, magnesium prevents the translocation of β-catenin into the nucleus, and this effect is not observed if transmembrane magnesium transport is abolished with the use of 2-APB. The inhibitory effect of magnesium on the cellular signaling pathway is reflected by a reduced expression of frizzled 3, VCAN/versican, cyclin D1 and c-Myc and enhanced expression of DKK-1. All this magnesium effects are reversed by 2-APB. The role of DKK1 in the inhibition of mineralization is clearly stablished. In a previous publication of our group [27], we have demonstrated that calcium deposition was significantly reduced when DKK1 was added to VSMC cultured with high phosphate.

Thus, our results show that high magnesium prevents the phosphate-induced Wnt/β-catenin activation. For the first time, this provides a possible mechanism by which magnesium reduces calcification in VSMC under calcifying conditions.

2-APB is an inhibitor of TRP (Transient Receptor Potential) receptor family [48], [49] including TRPM7 [17], Thus 2-APB inhibits the magnesium channel efficiently but it is not specific. Additional studies were performed to knock down TRPM7 expression and determine the role of TRPM7 on the regulation of Wnt/β-catenin signaling pathway. We found that the inhibition of 50% of gene expression results in activation of Wnt/β-catenin genes without producing any change in DKK1 expression. This suggests that the partial inhibition of TRPM7 is not enough to down-regulate DKK1, nevertheless, as we showed in results, the presence of moderately elevated magnesium concentration can significantly up-regulate this inhibitor of the Wnt pathway. Taken together these data reinforce the importance of magnesium and TRPM7 on regulation of Wnt/β-catenin signaling pathway.

Another novel and notable finding of the present work is the reduction of calcification in VSMC treated with magnesium after calcification has already been established. These experiments were performed to investigate whether magnesium is able to stop the progression of calcification. The results indicate that calcification is not only halted but even decreased once magnesium is added. This reversion of calcification is accompanied by significant changes in gene expression of Cbfa-1, osterix, MGP and OPG which further substantiates our results. Notably, the effect of adding magnesium is at least as strong as the effect of reducing phosphate levels for all parameters investigated.

An inhibition of the magnesium channel TRPM7 by 2-APB results in the same calcification level found at the starting point (5 days). This is accompanied by a marked down-regulation of MGP and OPG. Of note, Cbfa-1 and osterix are also partly down-regulated. Taken together, the inhibition of magnesium entrance into VSMC once calcification has been initiated does not completely abrogate but reduces the protective effect of magnesium.

These results open new perspectives for the treatment of already existing calcifications in patients and may imply that serum magnesium levels above the upper limit of normal might be beneficial in reducing vascular calcification. One could hypothesize that the administration of magnesium salts to renal patients might be beneficial through two independent mechanisms: firstly, the lowering of serum phosphate and secondly, a direct cellular effect of magnesium. To confirm the clinical relevance of these findings prospective, randomized, long-term clinical trials are needed to assess an independent role of magnesium on vascular calcification.

In conclusion, even only slightly increased magnesium concentrations not only prevent, but also reverse VSMC calcification in *vitro*. The effect of magnesium on VSMC calcification appears to be a result of the inhibition of Wnt/β-catenin signaling pathway. This novel finding provides an important insight into the cellular mechanisms by which magnesium exerts its beneficial effect on VSMC calcification.
